# Squamous differentiation in patients with superficial bladder urothelial carcinoma is associated with high risk of recurrence and poor survival

**DOI:** 10.1186/s12885-017-3520-1

**Published:** 2017-08-08

**Authors:** Gang Li, Jianpeng Yu, Hualin Song, Shimiao Zhu, Libin Sun, Zhiqun Shang, Yuanjie Niu

**Affiliations:** 1 Department of Urology, Tianjin Institute of Urology, The second hospital of Tianjin Medical University, Tianjin, 300211 China; 2 Department of Urology, First Hospital of Shanxi Medical University, Taiyuan, China

**Keywords:** Squamous differentiation, Urothelial carcinoma, Transurethral resection of bladder tumor, Radical cystectomy, Recurrence, Bladder cancer

## Abstract

**Background:**

The independent prognostic role of squamous differentiation in pT1 bladder urothelial carcinoma has not been reported in previous studies. This article describes the impact of squamous differentiation on tumor recurrence and survival, and whether this histologic variant could indeed alter definitive treatment, based on single center-based retrospective data.

**Methods:**

Totally, we retrieved (1)1449 histologically confirmed pT1 bladder urothelial carcinoma patients without histologic variants; (2)227 pT1 bladder urothelial carcinoma patients with squamous differentiation in our institution, from May 2004 to Oct 2015. The total amount of high/low grade urothelial carcinoma patients was 991/685 respectively. Transurethral resection of bladder tumor (TURBT) and intravesical chemotherapy were performed as initial treatments for all the patients. The clinical and pathological characteristics, treatment and survival outcomes were compared between squamous differentiation-positive and squamous differentiation-negative patients.

**Results:**

In our study, 14% urothelial carcinoma patients were detected with squamous differentiation. The mean age of all the patients examined was 66.4, of whom 82% were males. The 5-year cancer specific survival rates were 69% for squamous differentiation-positive patients and 91% for squamous differentiation-negative patients (*p* < 0.001). Recurrence proved to be more common in squamous differentiation-positive patients than in negative patients. In the results of the univariate and multivariate Cox proportional hazard analysis, tumor size, lymphovascular invasion, recurrence and squamous differentiation were confirmed to be the prognostic factors associated with patients’ survival.

**Conclusions:**

Squamous differentiation in pT1 bladder urothelial carcinoma is correlated to high risk of recurrence and poor prognosis as an independent prognostic factor. Radical cystectomy is essential for recurred high grade pT1 bladder urothelial carcinoma with squamous differentiation accompanied by lymphovascular invasion.

## Background

Approximately 380,000 bladder cancer patients are diagnosed and 150,000 patients die annually worldwide [1]. Urothelial carcinoma accounts for the majority of bladder cancers and has a substantial public health impact [2–4]. The morphological diversity of urothelial carcinoma is quite common. Squamous differentiation represents the most common form of mixed differentiation in invasive urothelial carcinoma and tremendous studies reveal that squamous differentiation may represent a precursor to invasive bladder cancer [5]. Squamous differentiation warrants aggressive therapy and may be beneficial for close monitoring [6]. Much attention has been paid to squamous differentiation in invasive bladder cancer [5, 7, 8], however, its significance on pT1 bladder tumor was ignored. Urothelial carcinoma with squamous differentiation is expected to be more aggressive, compared with pure bladder urothelial carcinoma without squamous differentiation. It is hypothesized that similar pathological stage pT1 urothelial carcinoma with squamous differentiation is more aggressive and greatly related to poor prognosis.

## Methods

We retrospectively analyzed 1676 bladder urothelial carcinoma patients between May 2004 and Oct 2015 in our institution. The pathologic stage was determined according to the 2009 Union for International Cancer Control (UICC) TNM staging system [9]. Pathologic grading of each tumor was based on the 2004 World Health Organization (WHO) classified system for non-invasive urothelial neoplasia [10]. The medical records and clinical data of the 1676 enrolled patients were reviewed. Patients with a history of previous urothelial carcinoma, other variant morphology, or concomitant upper tract urothelial carcinoma were excluded. TURBT and intravesical chemotherapy with Pirarubicin or camptothecin were performed as initial treatments for all the patients. Subsequent cystoscopy has been suggested to be performed during the postoperative follow-up period based on the European and US guidelines [11]. According to individual histological grade and stage, all recurrent tumors received re-TURBT or radical cystectomy. High grade recurrent tumors were suggested RC and low grade were proposed re-TURBT, however, the choices were based on the patients’ decisions. All of the enrolled patients were divided into two cohorts by squamous differentiation or not, and all patients with tumor recurrence were divided into two cohorts by TURBT and radical cystectomy. Age, gender, tumor grade, tumor multiplicity, tumor size, lymphovascular invasion, recurrence and squamous differentiation were assessed as prognostic factors. To avoid the influence of bladder tumor grade, analyses were also performed in low-grade and high-grade tumors with or without squamous differentiation respectively. Analyses of the prognostic implications of these factors were performed with the cancer specific survival rates. All demographic and pathological variables were queried. Variables were evaluated for inconsistencies and data integrity.

### Pathology

All surgical specimens determined by pathologists were submitted en bloc. Each of the pathology slides was evaluated case by case and assembled methodically for the adequate determination of grade and stage. Three representative slides of all the sections from each patient were assigned and re-reviewed independently by at least two pathologists in our institute to identify reported pathologic findings and squamous differentiation status. Presence of squamous differentiation, indicated by keratinization or intercellular bridges, was systematically assessed by hematoxylin and eosin (H&E) staining. The presence of lymphovascular invasion was also recorded using H&E staining. The criteria for confirming characterization of squamous differentiation did not change over the study period.

### Statistical methods

The period between the day of surgery and the day of bladder cancer specific death was considered as cancer specific survival. The independent-sample Student’s t-test and chi-square test were used to assess continuous variables and categorical variables, respectively. Overall survival trends and curves were calculated by the Kaplan–Meier method and differences were evaluated using the log-rank test. Univariate and multivariate analyses using the Cox regression models were performed to examine overall survival after operation. A *p* value of ≤0.05 (two-sided) was considered to indicate a statistically significant difference. All statistical analysis was performed with SPSS 22 statistical software (SPSS, IBM Corporation, Armonk, NY, USA).

## Results

### Clinical characteristics

Clinical and pathological characteristics were shown in Table 1. Average patient age was 66 years old. In this study, 1376 patients were males and 300 patients were females with a 5:1 male-to-female ratio. Of the 1676 patients, squamous differentiation was determined in 227 (14%) patients. Squamous differentiation was not significantly associated with age (*p* = 0.150), gender (*p* = 0.499) and tumor size (*p* = 0.708). However, high grade tumors with squamous differentiation were more common than high grade tumors without squamous differentiation (66% versus 58%, *p* = 0.022). The tumor multiplicity rate was significantly different between squamous differentiation-positive patients (115 patients, 51%) and squamous differentiation-negative patients (615 patients, 42%) (*p* = 0.02). Lymphovascular invasion rate showed a significant difference between the two cohorts, urothelial carcinoma with squamous differentiation revealed an increased occurrence of lymphovascular invasion (67 squamous differentiation-positive patients (30%) versus 121 squamous differentiation-negative patients (8%)) (*p* < 0.001). Recurrent patients with squamous differentiation was 72 (32%) and recurrent patients without squamous differentiation was 359 (25%) (*p* = 0.026). As data shown in Table 2, all recurrent patients underwent re-TURBT or radical cystectomy. According to decision of patients and our suggestions, 139 (61%) high grade recurrent patients were received radical cystectomy and 118 (58%) low grade recurrent patients underwent radical cystectomy.

**Table 1:** Association of squamous differentiation with clinical and pathological characteristics

Characteristic		Total	Squamous differentiation-negative	Squamous differentiation-positive	*p* value
Cases, n (%)		1676 (100)	1449 (86)	227 (14)	
Mean age (range),years		66.4 (24–92)	66.5 (26–92)	65.4 (24–90)	0.150
Gender, n (%)	M	1376 (82)	1186 (82)	190 (84)	0.499
	F	300 (18)	263 (18)	37 (16)	
	M:F	5:1	5:1	5:1	
Tumor grade, n (%)	Low	685 (41)	608 (42)	77 (34)	0.022
	High	991 (59)	841 (58)	150 (66)	
Tumor multiplicity, n (%)	No	946 (56)	834 (58)	112 (49)	0.02
	Yes	730 (44)	615 (42)	115 (51)	
Tumor size (cm), n (%)	<3	1089 (65)	939 (65)	150 (66)	0.708
	≥3	587 (35)	510 (35)	77 (34)	
Lymphovascular invasion, n (%)	No	1488 (89)	1328 (92)	160 (70)	<0.001
	Yes	188 (11)	121 (8)	67 (30)	
Recurrence, n (%)	No	1245 (74)	1090 (75)	155 (68)	0.026
	Yes	431 (26)	359 (25)	72 (32)	

*M* male; *F* female

**Table 2:** The patients of recurrence underwent re-transurethral resection of bladder tumor or radical cystectomy

	Tumor grade	Squamous differentiation-negative	Squamous differentiation-positive	Total
re-TURBT	Low	75 (21%)	9 (12%)	84 (20%)
	High	68 (19%)	22 (31%)	90 (21%)
Radical cystectomy	Low	113 (31%)	5 (7%)	118 (27%)
	High	103 (29%)	36 (50%)	139 (32%)
Total		359	72	431

*TURBT* transurethral resection of bladder tumor

### Oncological outcome

**Fig. 1:**
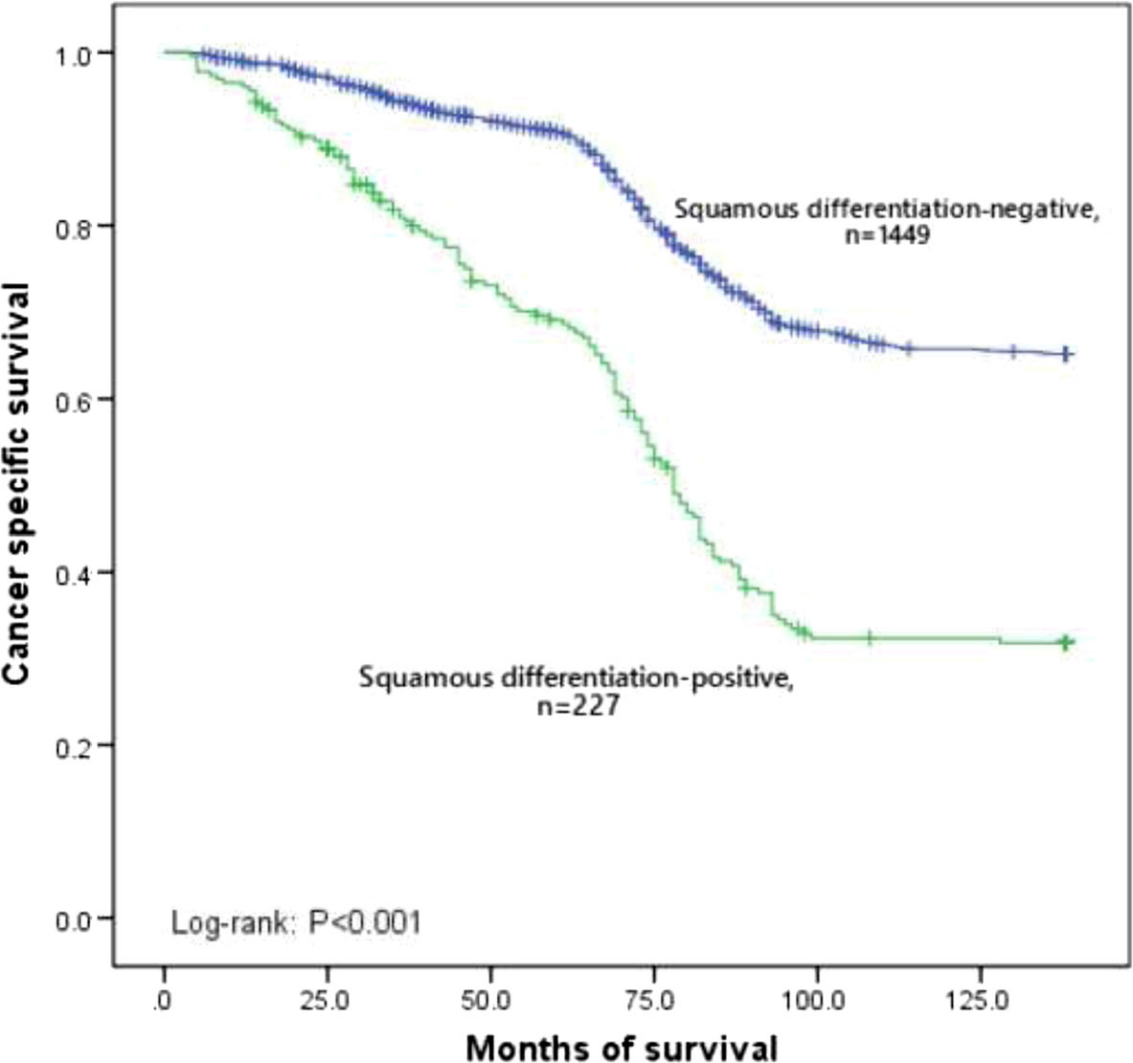
Kaplan–Meier curve of the cancer specific survival rates for the squamous differentiation-negative and squamous differentiation-positive (*p* < 0.001)

**Fig. 2:**
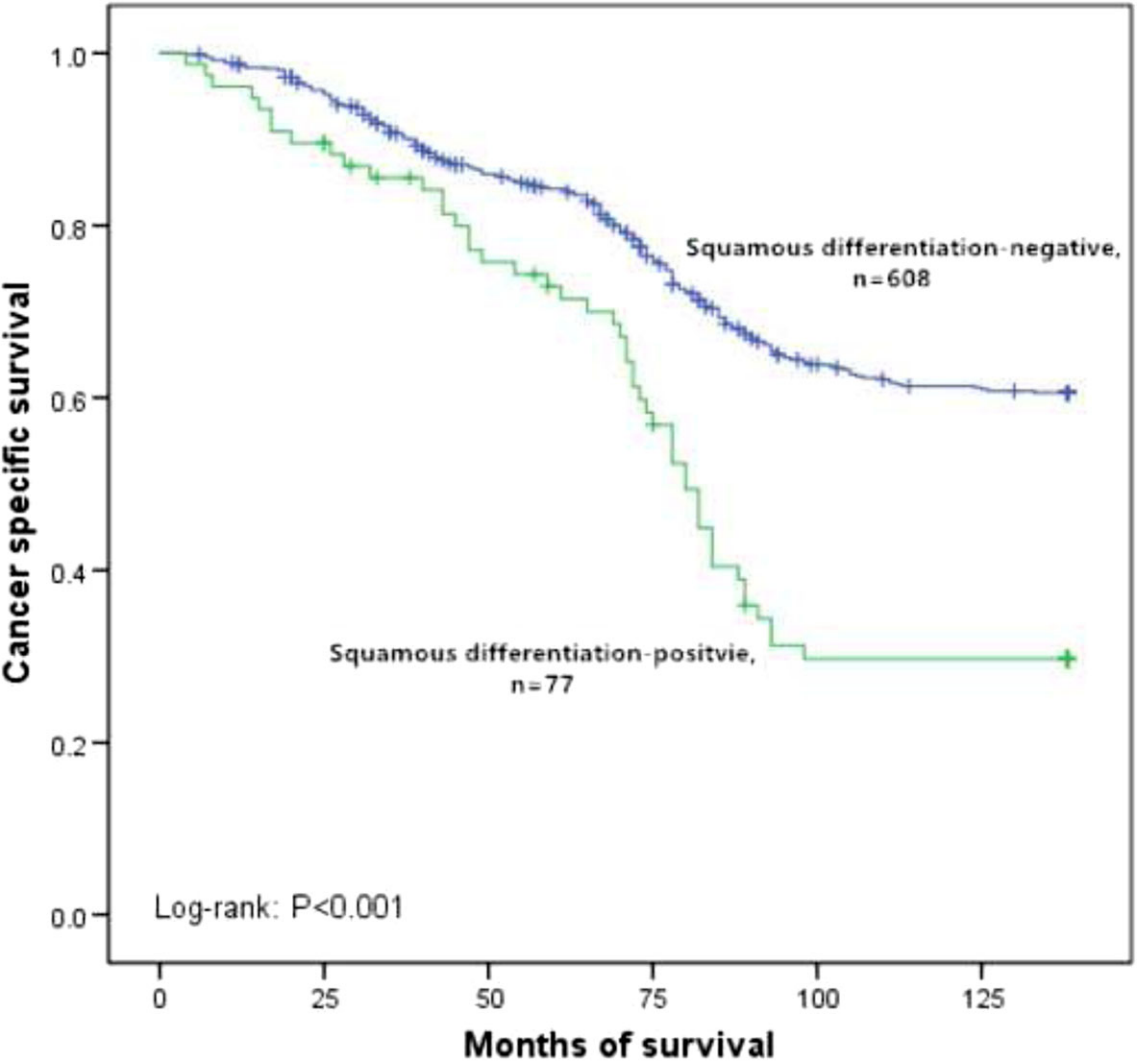
Kaplan–Meier curve of the cancer specific survival rates for the squamous differentiation-negative and squamous differentiation-positive in subgroup of low-grade (*p* < 0.001)

**Fig. 3:**
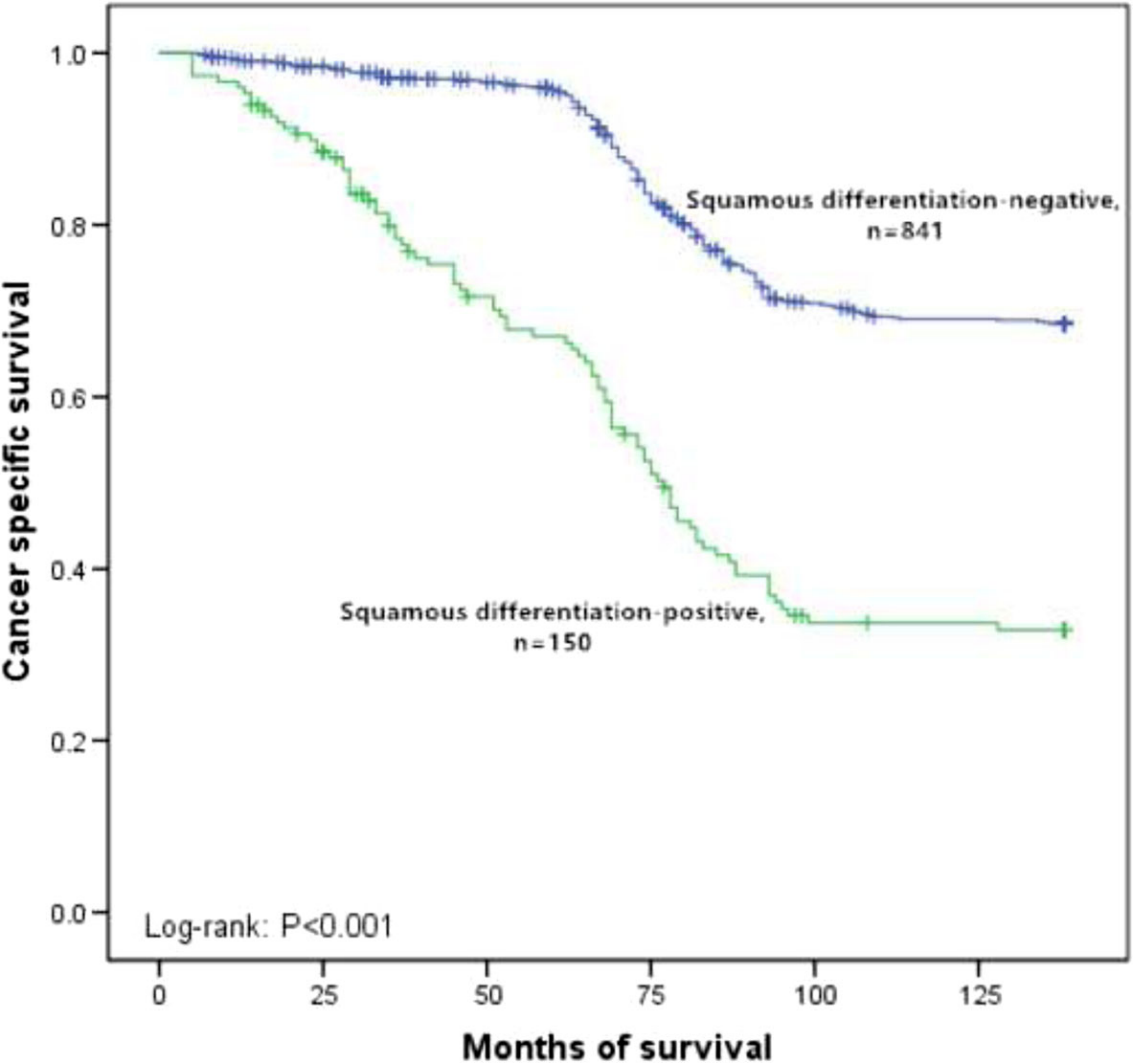
Kaplan–Meier curve of the cancer specific survival rates for the squamous differentiation-negative and squamous differentiation-positive in subgroup of high-grade (*p* < 0.001)

In this retrospective study, the median follow-up was 78 months (range 4 to 138). Because of cancer metastasis, 601 patients (36%) died during this follow-up period. The overall 5 year cancer specific survival rate was 88%. Furthermore, the 5 year cancer specific survival rates were 69% for squamous differentiation-positive patients and 91% for squamous differentiation-negative patients (*p* < 0.001, Fig. 1). In subgroup of low-grade, the 5 year cancer specific survival rates were 84% in squamous differentiation-negative patients and 73% in squamous differentiation-positive patients (*p* < 0.001, Fig. 2). In subgroup of high-grade, the 5 year cancer specific survival rates were 96% in squamous differentiation-negative patients and 67% in squamous differentiation-positive patients (*p* < 0.001, Fig. 3). Because the high grade recurrent patients received radical cystectomy were more than low grade recurrent patients, compared with low-grade squamous differentiation-negative patients, the 5 year cancer specific survival rate was a little higher in subgroup of high-grade squamous differentiation-negative patients (96% versus 84%). Recurrence was observed in 431 (26%) patients. However, recurrence was more common in squamous differentiation-positive patients than squamous differentiation-negative patients (32% versus 25%, *p* = 0.026). Cox proportional hazard analysis was used for further analysis (Table 3). In the results of univariate analysis, tumor size (HR 1.397, 95% CI 1.052–1.856, *p* = 0.021), lymphovascular invasion (HR 1.272, 95% CI 1.002–1.614, *p* = 0.048), recurrence (HR 5.726, 95% CI 4.707–6.965, *p* < 0.001), and squamous differentiation (HR 2.091, 95% CI 1.709–2.557, *p* < 0.001) were found to be the prognostic factors associated with cancer specific survival rates. Moreover, according to the results of the multivariate Cox proportional hazard analysis, tumor size (HR 1.312, 95% CI 1.107–1.556, *p* = 0.002), lymphovascular invasion (HR 1.291, 95% CI 1.021–1.631, *p* = 0.033), recurrence (HR 5.610, 95% CI 4.633–6.793, *p* < 0.001) and squamous differentiation (HR 2.068, 95% CI 1.693–2.526, *p* < 0.001) significantly influenced the cancer specific survival rates.

**Table 3:** Univariate and multivariate Cox regression analyses predicting cancer specific survival

Variable	Univariate analysis			Multivariate analysis		
	HR	95% CI	*P*	HR	95% CI	*P*
Age	1.003	0.997–1.010	0.339	——	——	——
Gender	1.039	0.847–1.275	0.714	——	——	——
Tumor grade	0.944	0.722–1.234	0.674	——	——	——
Tumor multiplicity	0. 900	0.688–1.178	0.444	——	——	——
Tumor size	1.397	1.052–1.856	0.021	1.312	1.107–1.556	0.002
Lymphovascular invasion	1.272	1.002–1.614	0.048	1.291	1.021–1.631	0.033
Recurrence	5.726	4.707–6.965	<0.001	5.610	4.633–6.793	<0.001
Squamous differentiation	2.091	1.709–2.557	<0.001	2.068	1.693–2.526	<0.001

*HR* hazard ratio; *CI* confidence interval

**Fig. 4:**
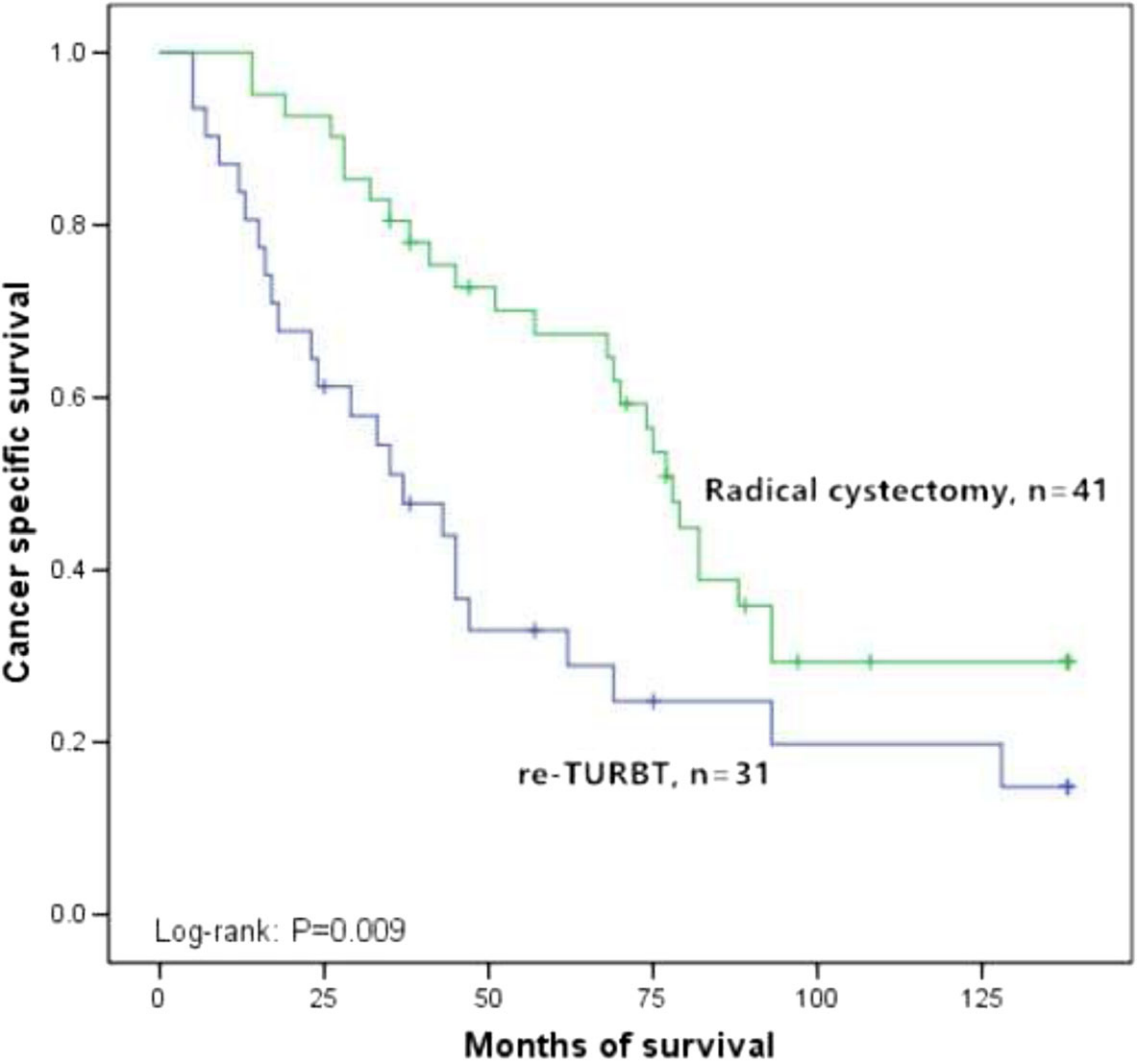
Kaplan–Meier curve of the cancer specific survival rates for re-transurethral resection of bladder tumor and radical cystectomy in the squamous differentiation-positive (*p* = 0.009). TURBT: Transurethral resection of bladder tumor

**Fig. 5:**
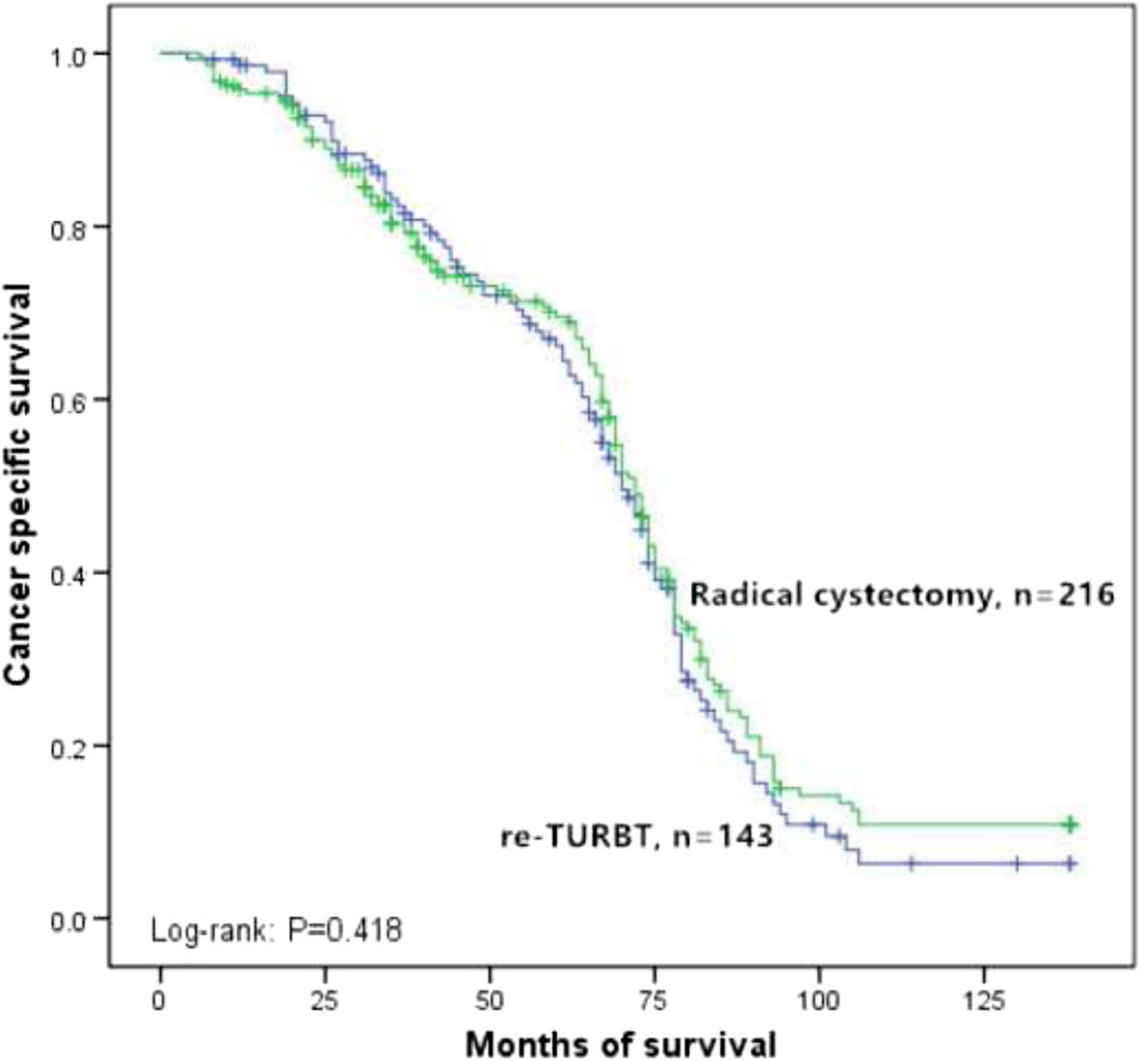
Kaplan–Meier curve of the cancer specific survival rates for re-transurethral resection of bladder tumor and radical cystectomy in the squamous differentiation-negative (*p* = 0.418). TURBT: Transurethral resection of bladder tumor

After recurrence stratified by re-TURBT and radical cystectomy in squamous differentiation-positive group and squamous differentiation-negative group, cancer specific survival rates were estimated by the Kaplan–Meier analysis. Patients after re-TURBT had shorter median cancer specific survival than those underwent radical cystectomy in squamous differentiation-positive patients (33 versus 67 months, *p* = 0.009, Fig. 4). Nevertheless, no significant imbalance was found between re-TURBT and radical cystectomy groups in squamous differentiation-negative patients (66 versus 70 months, *p* = 0.418, Fig. 5).

### Pathology

**Fig. 6:**
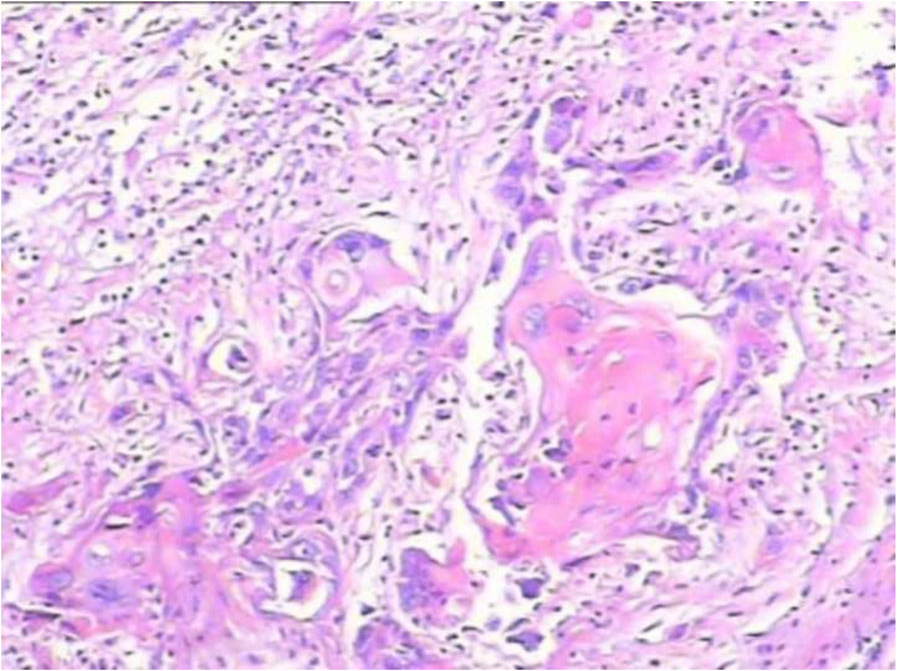
The component of tumor was considered to be squamous when intercellular bridges and/or keratinization were evident

**Fig. 7:**
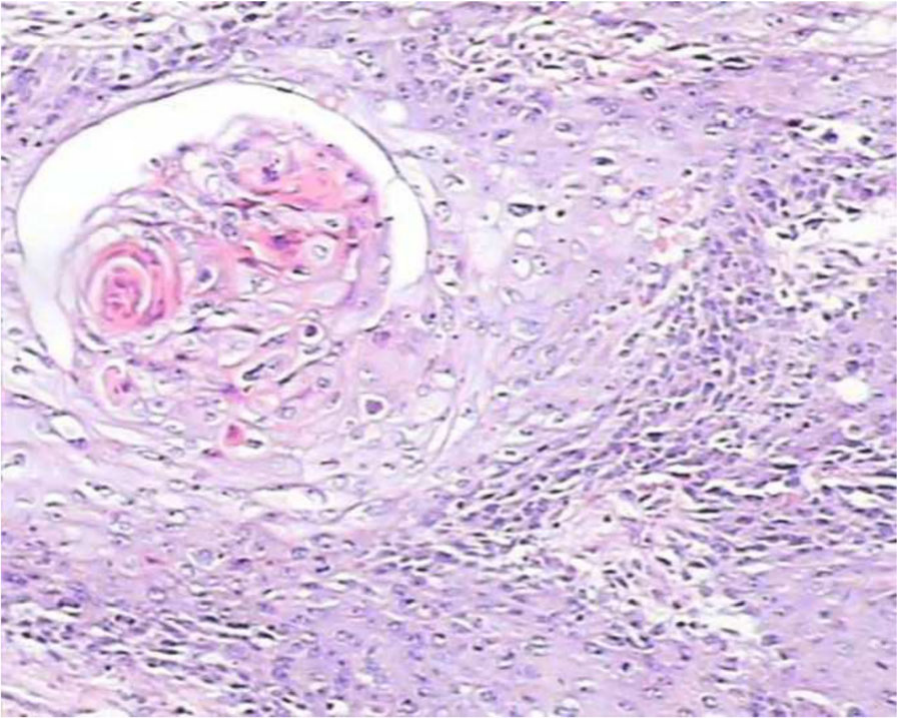
Stained sections in hematoxylin and eosin were used to evaluate the presence of lymphovascular invasion. H&E: hematoxylin and eosin

Pathologists determined and confirmed 227 squamous differentiation patients. Evident intercellular bridges and/or keratinization indicated the presence of squamous differentiation (Fig. 6). The main histological characteristic is: 1. Presence of large and medium sized nucleolus in strands or nests of infiltrating tumor cells; 2. without clearly separated amphophilic or eosinophilic cytoplasmic background. The presence of lymphovascular invasion was determined by the stained sections in H&E (Fig. 7).

## Discussion

Prior studies well established that urothelial carcinoma harbors remarkable propensity for divergent differentiation [12–14]. Squamous differentiation has been suggested to be the most common histological variation in bladder urothelial carcinoma and it may present basaloid or clear cell features [15]. However, mechanisms of squamous differentiation in urothelial carcinoma is not very clear. In 1925, Wolbach and Howe found that long time vitamin A deficiency treatment could lead to bladder squamous differentiation in rats [16]. More recently, several findings together with previous studies on the development of squamous differentiation under condition of retinoid deficiency, strongly recommend that retinoid signaling pathways and related networks are significant in the development of squamous lesions in the urinary bladder [17–20]. Maraver et al. showed that downregulation of NOTCH signaling contributed to occurrence of aggression in squamous lesions [21, 22]. Some transcriptional regulators (like forkhead boxA1) and activation of epidermal growth factor receptor (EGFR) pathway also play key roles in squamous differentiation in urothelial carcinoma. Moreover, Guma et al. found that HPV infection was associated with urothelial carcinoma with squamous differentiation [23]. Fujii et al. proposed that miR-145 may promote stem cell-like transformation in urothelial carcinoma cells and lead to differentiation into multiple cell types [24]. Therefore, it still requires a concerted effort by the research communities to differentiate “drivers” from “passengers” in the mechanisms of squamous differentiation.

Although thorough and careful light microscopic assessment is the best way to determine squamous lesions, it could be sometimes difficult to differentiate squamous differentiation from conventional urothelial carcinoma or squamous cell carcinoma, particularly in patients with borderline features. In such situation, the use of immunohistochemical technique, in addition to light microscopy, may be helpful. Huang et al. recently reported that MAC387, desmoglein-3 and TRIM29 could be a novel panel of specific markers for squamous differentiation in a series of bladder cancer [25]. In our study, the tumors showed a not clearly separated eosinophilic or amphophilic cytoplasmic background and strands or nests of infiltrating tumor cells with large and medium sized nuclei, often with nucleolus. Stained sections in H&E were used to evaluate the presence of squamous differentiation.

Many of recent studies have suggested the clinical significance of squamous differentiation in urothelial carcinoma. Fikret et al. evaluated the data from 223 patients treated with transurethral resection for bladder cancer and found squamous differentiation type of bladder urothelial carcinoma was more aggressive than conventional urothelial carcinoma and squamous differentiation may lead to poor prognosis [15]. Gluck et al., who analyzed 361 patients with invasive bladder carcinoma, concluded that squamous differentiation carcinomas were more aggressive with worse prognosis than pure urothelial carcinoma [5]. Alberto et al. retrospectively selected 113 patients with transitional cell carcinoma and suggested that squamous differentiation was an independent prognostic factor for cancer specific survival in patients treated with radical cystectomy [9]. However, there is short of data on the significance of squamous differentiation in 1676 patients with pT1 bladder urothelial carcinoma. Our study showed further evidence suggesting that squamous differentiation, a pathological variant, was an independent prognostic indicator for pT1 urothelial carcinoma in bladder cancer. Although survival of recurred patients with invasive bladder cancer has been reported, there is little data on the survival of the recurred pT1 bladder cancer patients after re-TURBT or radical cystectomy. Our series found that patients after re-TURBT had shorter median cancer specific survival than those underwent radical cystectomy in squamous differentiation-positive group (33 versus 67 months, *p* = 0.009). Nevertheless, no significant difference was found between the two operations in squamous differentiation-negative recurred patients (66 versus 70 months, *p* = 0.418). We suggested surgeons should operate radical cystectomy routinely in recurred pT1 urothelial carcinoma with squamous differentiation.

Tang et al. retrospectively analyzed the data of 687 upper tract urothelial carcinoma patients and found that patients with squamous and/or glandular differentiation were more likely to receive aggressive tumor biological characteristics and tend to have worse postoperative outcomes [26]. Our series found that squamous differentiation (HR 2.091, 95% CI 1.709–2.557, *p* < 0.001) was an independent prognostic factor associated with cancer specific survival in both univariate analysis and multivariate analyses. Tumor size (HR 1.397, 95% CI 1.052–1.856, *p* = 0.021), lymphovascular invasion (HR 1.272, 95% CI 1.002–1.614, *p* = 0.048) and recurrence (HR 5.726, 95% CI 4.707–6.965, *P* < 0.001) were also found to be the prognostic factors associated with cancer specific survival. In addition, in the results of the multivariate Cox proportional hazard analysis, tumor size (HR 1.312, 95% CI 1.107–1.556, *p* = 0.002), lymphovascular invasion (HR 1.291, 95% CI 1.021–1.631, *p* = 0.033), recurrence (HR 5.610, 95% CI 4.633–6.793, *p* < 0.001) and squamous differentiation (HR 2.068, 95% CI 1.693–2.526, *p* < 0.001) significantly influenced the cancer specific survival too.

It is routine to perform radical surgery on muscle-invasive tumor with urothelial carcinoma with squamous differentiation. However,if the patient is a non-muscle invasive case, it will be a dilemma in treatment option. Immunotherapy with bacillus Calmette–Guerin (BCG) on patients with variant tumors has been reported rarely [27]. Gofrit et al., who focused on 41 Ta or T1 patients diagnosed by second biopsy and received immunotherapy with BCG, found that BCG treatment was risky for patients with non-muscle invasive variant bladder tumors and these patients were more liable to worsen [28]. Furthermore, compared with conventional high-grade tumors, successful salvage may be a big challenge for these patients [28]. In this study, TURBT and intravesical chemotherapy with Pirarubicin or camptothecin were performed as initial treatments for all the patients to avoid adverse clinical course. Previous studies reported that intravesical BCG had worse prognosis, which was consistent with our study. According to previous results and our series, it is suggested that radical cystectomy is necessary for recurred pT1 urothelial carcinoma with squamous differentiation patients.

## Conclusion

As it is concerned, the study is the first to concentrate on clinical significance of squamous differentiation on pT1 bladder urothelial carcinoma. When treated as an independent prognostic factor, squamous differentiation is related to higher risk of recurrence and poorer prognosis on patients: Suggesting its unique clinically-related nature. Based on the results mentioned above, radical cystectomy is of huge essence to deal with recurred patients suffering from high grade urothelial carcinoma with squamous differentiation, especially with lymphovascular invasion.

## Abbreviations

CI: confidence interval
EGFR: epidermal growth factor receptor
H&E: hematoxylin and eosin
HR: hazard ratio
TURBT: Transurethral resection of bladder tumor
UICC: Union for International Cancer Control
WHO: World Health Organization
